# Political change as group-based control: Threat to personal control reduces the support for traditional political parties

**DOI:** 10.1371/journal.pone.0278743

**Published:** 2022-12-08

**Authors:** Álvaro Rodríguez-López, Soledad de Lemus, Marcin Bukowski, Anna Potoczek, Immo Fritsche

**Affiliations:** 1 Mind, Brain and Behavior Research Center (CIMCYC), University of Granada, Granada, Spain; 2 Department of Philosophy, Institute of Psychology, Jagiellonian University, Kraków, Poland; 3 Interdisciplinary Centre for Mathematical and Computational Modelling, University of Warsaw, Warsaw, Poland; 4 Wilhelm Wundt Institute for Psychology, Leipzig University, Leipzig, Germany; University of Connecticut, UNITED STATES

## Abstract

People desire agentic representations of their personal and collective selves, such as their own nation. When national agency is put into question, this should increase their inclination to restore it, particularly when they simultaneously lack perceptions of *personal* control. In this article, we test this hypothesis of group-based control in the context of political elections occurring during socio-economic crises. We propose that people who are reminded of low (vs. high) personal control will have an increased tendency to reject traditional political parties that stand for the maintenance of a non-agentic political system. We experimentally manipulated the salience of low vs. high personal control in five studies and measured participants’ intentions to support traditional and new political parties. Across four of five studies, in line with the predictions, low personal control reduced support for the main traditional conservative party (e.g., Partido Popular (PP) in Spain, the Republicans in France). These results appeared in contexts of national economic and/or political crisis, and were most pronounced when low (vs. high) national agency was made salient in Studies 4 and 5. The findings support the notion that rejecting the stability of the national political system can serve as a means to maintain a sense of control through the collective self.

## Introduction

In the last decade, large sociopolitical changes have taken place worldwide. In political terms, the rise of right-wing and populist movements has been discussed as an effect of the 2008 economic crisis [1]. This socio-economic crisis affected not only people’s life conditions (e.g., unemployment, increased social and economic inequality, reduced income) but also evoked psychological threats to basic motives of control and predictability [2, 3]. Economic threat was shown to motivate psychological responses in order to maintain or restore a sense of control and to activate personal or collective attitudes and responses that are palliative (e.g., blaming, prejudice) or socially constructive (e.g., collective action toward social change) [4]. In this research, we focus on the psychological underpinnings of changes in support for traditional system-affirming parties as a function of people’s low personal control. We argue that in the context of strong economic and social threats in the last decade in Europe, traditional parties that may have contributed to a lack of perceived agency at the national level could promote political distancing of voters, as a form of coping with threatened control.

Personal control has been defined as the extent to which a person can produce desired outcomes and prevent undesired ones [5] and it is considered to be a basic human need [6, 7]. When personal control is threatened, people are motivated to reestablish the belief that the world is controlled through their autonomous self [8, 9]. According to group-based control theory [8], this applies to both representations of the self as an individual person (e.g., as “I”) and definitions of the self as a collective agent (e.g., as “We”; *social identity*) [10, 11]. Accordingly, people desire control, or agency, for their own self (“I”) as well as for their self-defining ingroups (“We”). Also, when people’s sense of personal control is threatened they take efforts to restore their sense of control on the level of their social self by identifying with agentic ingroups [12] or engaging in group-based action in order to re-establish a sense of control through their (social) self [13].

Some situations can threaten this perception of personal and group-based control on a large scale. The 2008 economic crisis [3], or recently the crisis generated as a result of the COVID-19 pandemic [14] are good examples of threats to personal and collective control. Several studies have shown that the 2008 economic crisis had a great impact on well-being [15] and threatened people’s sense of control [4]. Such threats to control elicited control-restoring responses which might operate at the ingroup level (e.g., increased ingroup trust) or intergroup level (e.g., promoting collective actions for social change when economic threat is salient) [4]. The purpose of this research is to examine the effect of low personal control on voting intentions in the context of an economic and social crisis. We assume that voting can function as a direct coping mechanism with lack of control experiences [3, 13]. Recent research has shown that following general voting norms of the ingroup is an effective way to cope with lack of sociopolitical control [16]. Extending this work, we focus on whether personal control threat can influence people’s voting intention by reducing support for traditional parties. Specifically, we argue that rejecting the stability of the national political system, and thus indirectly supporting collective change, is a means to restore a sense of control through the collective self.

### Coping with threats to control in a sociopolitical context

A socio-economic crisis can guide people to take different steps to restore personal control, from individual and self-contained measures to social strategies at the group level, producing changes in people’s social behaviors, values and attitudes [3, 13]. The 2008 economic crisis strongly hit Southern European countries such as Spain, Greece or Italy, provoking a massive increase in unemployment rates amongst the youth, and generally enlarging social and economic inequalities. Such economic threats fostered hostile interethnic attitudes but also increased ingroup trust, group efficacy and citizens’ support for collective actions when national economic identity was salient in the context of the economic recession in Spain [4]. Moreover, lack of control triggered by the economic crisis also led to attributing blame to specific groups (e.g., bankers or political parties) in order to restore perceived control over the situation in the Spanish context [2]. If established political parties are blamed for threatening people’s control at the national level (e.g., through austerity policies), this should reduce people’s support for them, particularly when they are highly motivated to engage in group-based control [4].

Following a group-based account, we argue that threats to personal control in a national, political and economic context may encourage people to respond in a way that, in their opinion, re-establishes national agency [4]. For instance, Spanish people respond to subliminal cues that threaten their national identity, legitimizing the economic disadvantage by increasing their ingroup bias [17]. Group-based control research proposes, and has shown, two different collective responses to threatened personal control. First, people identify more strongly with salient social ingroups, especially when these are agentic [12]. Second, they more strongly pursue salient ingroup norms [13, 16] and ingroup goals (e.g., helping in the campaigns of a political ingroup) [18]. Importantly, such responses of ingroup support are assumed to be most pronounced when the agency of a potentially agentic ingroup simultaneously seems to be at stake [4, 9]. Threat to personal control should motivate people to re-establish control of a potentially agentic ingroup. We propose that threats to control experienced in the context of a socio-political crisis might cause traditional, system-affirming parties to be rejected. These parties can be perceived as threatening collective agency, thus people with low personal control can be motivated to stop supporting them as a way to restore their control and group agency.

This prediction presumably contradicts the conservative shift hypothesis according to which different threats may promote support for conservative ideologies as an attempt to decrease fear and anxiety [19]. Additionally, it opposes notions of the compensatory control model, which argues that when personal control is threatened, people try to preserve a sense of order by defending the legitimacy of different sociopolitical institutions [20]. To integrate the theories of group-based control and compensatory control [8, 21], it has been proposed that group-based control represents people’s primary response to threatened personal control. Only when collective routes to control seem futile do people resort to reducing uncertainty and, thus, rejecting change. According to group-based control theory, threats to personal control will provoke a motivated group-based shift [13, 22] e.g., by acting as group members and supporting collective actions performed by a relevant ingroup [23]. The model implies that when social change represents ingroup norms or serves the establishment of collective agency, people will more strongly pursue change under conditions of threatened personal control [13].

In sum, in times of threat, people might not support any system or party per se, but choose those that stand for (restoring) national agency. This may go along with people supporting parties that pursue change when this change promises to re-establish national agency, but rejecting parties that stand for preserving the status quo. When people perceive the current political system as non-agentic, striving for change might indicate an effort of group-based control. This might well be the case in countries that strongly suffered the consequences of the 2008 economic crisis and struggled to overcome them. In such socio-political contexts, several social movements emerged that demanded social change (e.g., los Indignados in Spain or Occupy Wall Street in the U.S.).

Beyond helping to restore national agency, identifying with, and supporting those movements might have intrinsic value for people who feel their control is threatened. This is because collectives who demand change might be perceived as being more agentic than collectives that just want to maintain the status quo. Demanding system change may mainly elicit internal causal attributions, suggesting goal-directed collective action can be an effective expression of collective agency. Instead, when groups demand system maintenance, this can also be attributed to many non-agentic causes, such as tradition, norm conformism, habits or even external pressures from international institutions (e.g., World Bank, EU). In line with this reasoning, Stollberg et al [13]. found that threat to personal control increased people’s conformity to salient ingroup norms when these were framed as a collectively shared demand for change but not when framed as a demand for stability. Applied to political decisions, this means that control threats experienced during global economic crises may foster people’s striving for collective social change. We argue that such change can be achieved, for instance, by not supporting system-affirming parties. We test this prediction, that low personal control can promote social change, in the form of reduced support for traditional, system-affirming parties in the aftermath of the 2008 economic crisis in two European countries: Spain and France.

### Overview of the present research

In the present article, we hypothesize that in the context of a general political crisis, people may reduce their support for system-affirming traditional parties when they are motivated to bolster their sense of control because personal control is threatened. We examine two factors that might moderate this effect, namely the perception of national agency and whether political and social change seem possible (e.g., reality constraints). We used the natural context of elections and political changes occurring in Spain (2015, 2016 and 2017) and France (2017), and also experimentally manipulated agency and stability in a hypothetical election scenario in the last study. Thus, we expect backlash against traditional parties to be a function of threat to personal control, mainly when national agency is threatened and when there are realistic chances for change to happen. By traditional parties, we refer to system-affirming parties that endorse more traditional values and also those whose speech is based on the defense of national and cultural traditionalism [24] (e.g., Partido Popular in Spain and Les Republicains in France).

We hypothesized that reminding participants of low personal control, especially in the context of economic and political crises that reveal a low level of group agency in current political solutions, will decrease their support for traditional parties. To test this hypothesis, we carried out 5 studies. Studies 1–3 and 5 were conducted in Spain, whereas Study 4 was conducted in France.

The 2008 economic crisis had a strong impact on Spanish citizens, increasing unemployment, poverty and social inequalities [25, 26]. Its effects led to high dissatisfaction with the government amongst Spaniards [27]. Discontent about the existing political system created space for new movements–e.g., in 2014 a new political party (Podemos) emerged and received 8% of the votes in the European elections. One year later, it became the third political force in Spain. Study 1 was carried out in Spain in 2015 when, for the first time, new parties that appeared as a reaction to the political crisis ran in the regional elections, breaking down the bipartidism system. In Study 2, we tested our predictions before the general national Spanish elections of 2016. In order to check whether the effects depended on the perceived agency of the group, we manipulated collective agency and control orthogonally in Study 3. Although group-based control theory would predict that the effects of low control on supporting traditional parties should appear more clearly when agency is low, this should not be the case when change does not seem possible. The sociopolitical context in Spain at that time impeded actual change. The third study was conducted after the second round of Spanish general elections in October 2016 when the country was under a period of political impasse with no party being able to form a government. That was a reality constraining situation that may have led people to search for stability rather than change. For this reason, in Study 4, we tested our predictions in a context in which the possibility for political change was less constrained, in France after the 2017 presidential elections in which a new leader had arisen (e.g., Emmanuel Macron) to the detriment of the two established parties (Les Republicaines and Parti Socialiste). Finally, we experimentally tested whether the perceived efficacy of stability versus change strategies determined the impact of threats to control under low agency conditions in Study 5, in a hypothetical election scenario in Spain in 2017. This article is framed in a chronological way with the aim to examine how personal control and group agency influenced political changes as a function of the specific sociopolitical context that followed the 2008 crisis in two different European countries.

## Study 1

We conducted Study 1 to examine whether threat to personal control affects attitudes towards and support for the different political parties in Andalusia, Spain, in 2015. We expected that in low control conditions, the main opposition traditional party and the main party in the government would receive relatively less support than in the high control conditions. At the time when the study was conducted, the socialist party in the government (PSOE) had been ruling for 37 years at the regional level. Thus, although this party does not represent traditionalism in terms of social values, it was representative of a well-established political system in the region. The main opposition party (PP, more traditional in values and economy) had never ruled in the region since democracy was established in Spain, but at that time it was the ruling party at the national level. Two new parties were running for the first time in these elections: Podemos (left wing) and Ciudadanos (liberal). As a consequence of reduced support for the system-affirming parties, we could expect to find increased relative support for the new parties under low control conditions.

### Materials and methods

All the studies received approval from the institutional research ethics committee of the University of Granada and the Public Health Code of France. All participants gave informed written consent in accordance with the Declaration of Helsinki.

#### Participants and design

Spanish nationals (50 male, 106 female), aged *M* = 27.14 (*SD* = 10.47), completed the experiment online via Qualtrics. The sample was completed using a snowball procedure through different social networks. The study followed an experimental unifactorial design with 3 conditions: high control, low control, and a null condition (where control was not manipulated). Participants were randomly assigned to one of the three conditions.

We excluded 10 participants who refused to fill in the manipulation task and those with missing values in the focal variables, which left us with a final sample of 146 participants. We conducted a sensitivity analysis using G*power [28]. Results showed that with this sample size (*N* = 146), the minimum effect size that we can detect for *α* = .05 and 1-*β* = .80 is *f* = .26 (minimum detectable effect).

#### Procedure

The study was introduced as a “questionnaire to learn about the personal experiences and opinions of different aspects of social life of Spanish citizens” and was administrated one week before the local elections that were held in March 2015 in Andalusia. First, participants completed the Need for Closure Scale [29], which was not included in the analyses (see Appendix 2 in the S1 File), and then they completed the manipulation and answered the variables as described below.

*Control manipulation*. Participants read a short report indicating experts’ opinion about the economic crisis, showing that it had a controllable (vs. not controllable) course and that its effects could be reduced (or not). Then, participants had to think about and write down 2 controllable (vs. non-controllable) effects of the economic crisis. This manipulation was previously used in other research that was performed in the context of the economic crisis in Spain [2] In the null condition, participants read a piece about sports (see Appendix 1 in the S1 File for all the condition details). Then, participants had to write down two examples of sports they practice in their daily life.

#### Measures

*Perceived control*. We asked participants to indicate on a 7-point scale (from 1 = Not at all, to 7 = Absolutely): *To what extent does the crisis cause you to lose control over your life*? *To what extent do you think you have control over the impact that the crisis has on your life*? *To what extent do you feel that you have control over your life at this moment*? Since the reliability of the 3 items of the scale was unacceptable (*α* = .33), and the correlations between items were low (Items 1–3: (*r* = -.360, *p* < 0.01; Items 1–2: *r* = .054, *p* = .507; Items 2–3: *r* = .201, *p* = .012), we consider the items independently because they address different aspects of control.

*System justification*. Participants rated their willingness to support the system using eight items on a 7-point-scale (from 1 = Strongly disagree to 7 = Totally Agree; *α* = .68), e.g., *In general*, *the Spanish political system works as it should* [30].

*Support for political parties*. We measured support for parties with three items referring to the affective response towards the different political parties in Spain *(How close do you feel to the proposals of the following parties*?), their intention to vote for each party in the next elections (*What is the chance you would vote for each of the following parties in the next election*?*)*, and their beliefs about the efficacy of each party to solve the economic situation if they were elected *(To what extent do you think that the management of each of the following parties*, *if it won the elections*, *would improve the socio-economic conditions in Spain*?). Participants gave their answers on a slider ranging from 0 to 100. We averaged across three items to create an index for each party (*α* = .93 to .95).

### Results

#### Perceived control

We did not find a significant effect of the control manipulation on items measuring perceived general control over life, all *F*s ≤ 1.08, ns.

#### System justification

We obtained a significant effect of threat to personal control manipulation on system justification in the ANOVA, *F*(2,146) = 3.40, *p* = .036, *η*^*2*^ = .05. Pairwise comparisons showed that in the high control (*M* = 3.31) condition participants support the system more than in the low control condition (*M* = 2.93) (*p* = .030; *d* = .52), while neither the low control (*p* = .47; *d* = .26) or the high control (*p* = .44; *d* = .27) were statistically different from the baseline condition (*M* = 3.11).

#### Support for the parties

**Fig 1:**
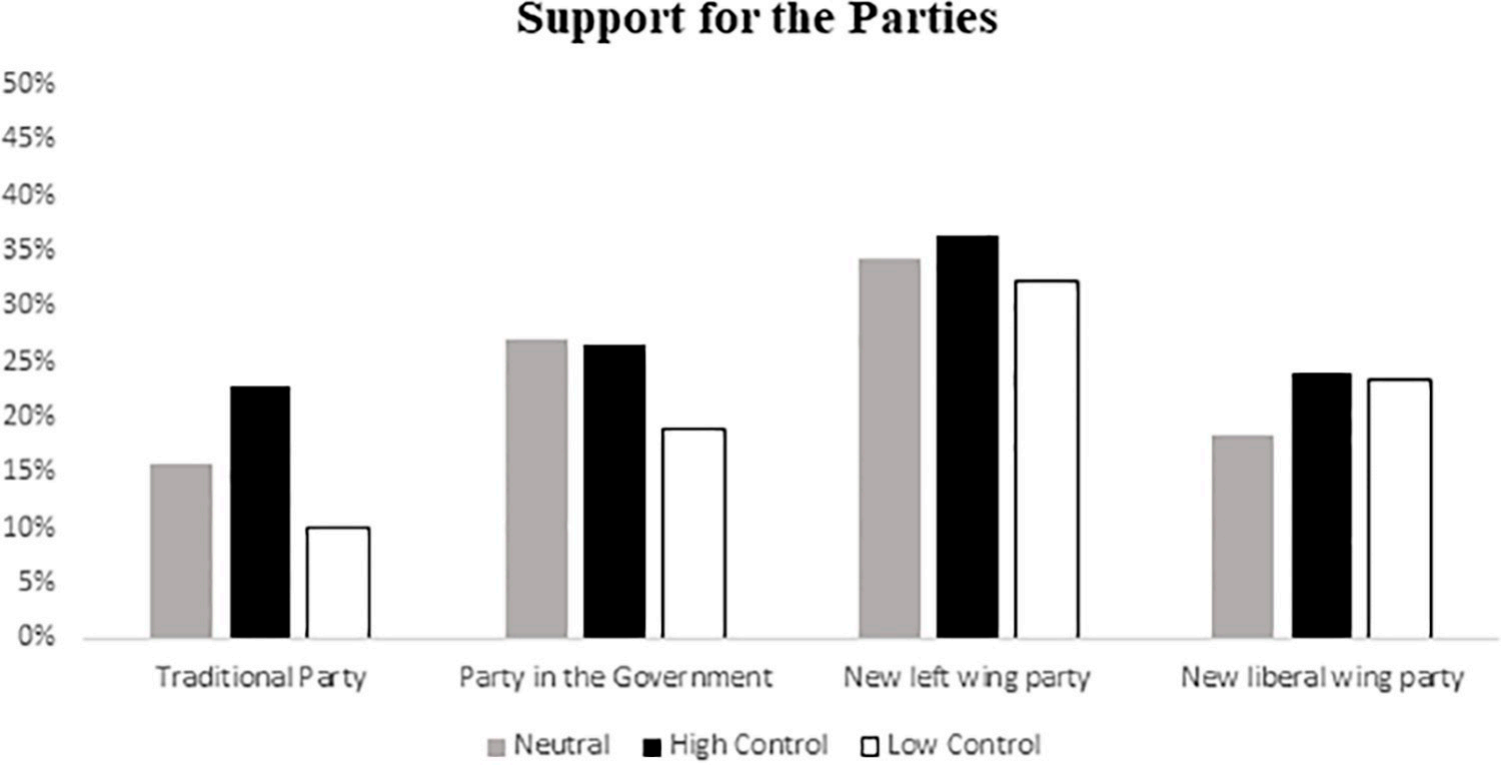
Support for the different political parties by experimental conditions (Traditional party: PP).

We expected that low control conditions would decrease support for system-affirming parties. We found an effect of threat to personal control manipulation on support for the traditional opposition party, *F* (2, 146) = 3.17, *p* = .045, *η*^*2*^ = .04. In line with our reasoning, pairwise comparisons show that participants supported the traditional party less in the low control condition (*M* = 10.01) compared to the high control condition (*M* = 22.83), *F*(2, 146) = 3.17, *p* = .038, *η*^*2*^ = .04. Although means were in the expected direction, the comparison between the low control and the baseline condition (*M* = 15.68) was non-significant, (*p* = .07 *d* = .26). There were no significant univariate effects of control for any other party, all *F* < 1.5, ns. (See Fig 1).

Regarding the correlational analyses, both participants’ perception that the crisis reduced their control (*r* = -.26, *p* = .005) and their perceived personal control (*r* = .26, *p* = .001) were significantly related to support for the traditional party but there was no relation between the impact of the economic crisis on the lives of the participants (*r* = .02, *p* = .78). That is, the less personal control they felt, and the more they perceived that the crisis affected their feelings of control, the less they supported the traditional opposition party.

### Discussion

The results of Study 1 partly confirm our hypothesis that participants in a low control condition will show less support for traditional parties. The main traditional opposition party was supported less by participants in the low control than in the high control condition. However, there was no significant difference between the low control and the null condition, so we cannot conclude that the effect is uniquely due to lack of control (and not to salient *high* control). Although the direction of the means was the same for the Socialist party that held the regional government at the time, this effect was not significant. The fact that the framework of the economic crisis referred to the country might have shifted participants’ attention toward the traditional party that was ruling at the national level. Low personal control reduced participants’ intention to vote for and support the traditional party compared to high control. Although we could not show a reliable control threat effect when comparing it to the neutral control group (null condition), the direction of the former effect suggests that one way to restore control at the group level implies supporting change by *not* supporting the traditional party. The manipulation of personal control threat did not influence general feelings of control over one’s life, but possibly activates the idea that the economic effects of the crisis were (un)controllable and triggers the search for causal attributions which could imply blaming the ruling parties, in line with previous studies using the same type of experimental manipulation [2]. Interestingly, we also found that participants under low control decreased their support for system justification claims, showing that their general motivation to maintain the status quo was affected by the threat to their personal control. This is in line with group-based control theory, indicating that participants do not support the system as a tool to reduce uncertainty (as compensatory control theory would predict), rather they search for a way to strengthen their group agency [3, 9].

## Study 2

The results of Study 1 suggest that in situations of threat to personal control, there is a decrease in support for the traditional party. We carried out a second study in order to corroborate our results. We expected that low control will predict less support for the traditional party that ruled in Spain at that time (Hypothesis 1). We also manipulated political efficacy to test whether the effects of control on support for the parties depended on perceived general political efficacy [31].

Study 2 was carried out in the framework of the second Spanish general elections that were run in 2016. After a first round of elections, when no political party received sufficient support to form a government, and the elected parties were unwilling to reach a coalitional agreement, the elections had to be repeated after six months.

The Spanish electoral system implies that the representation of parliamentary seats does not correspond to the actual number of votes that each party receives, which favored the traditional parties against the new political parties in the previous round of elections [32]. We measured participants’ support for a legislative change towards a proportional representation of the parties in the parliament based on actual votes. In line with our general hypothesis, we predicted that low control will increase support for such a legislative change (Hypothesis 2).

### Materials and methods

#### Participants and design

Spanish nationals (28 male, 60 female), aged *M* = 22.41 (*SD* = 3.88), were randomly assigned to one of 4 experimental conditions, following a 2 control salience (high/low) x 2 political efficacy (high/low) design. However, the small sample size prevented us from conducting interaction analyses, therefore we focus on a unifactorial design with control as a main predictor and controlling for the political efficacy manipulation as a covariate (the analyses with political efficacy as an orthogonal predictor can be found in the Appendix 2 in the S1 File). A sensitivity analysis for a one-way ANCOVA showed that with this sample size (*N* = 88), the minimum effect size that could be detected for *α* = .05 and 1-*β* = .80 is *f* = .30 (minimum detectable effect). No exclusions were needed based on the same criteria as in Study 1.

#### Procedure

The study was carried out on the 15th of June 2016, 11 days before the elections. Participants were recruited from the campus libraries of one Spanish university. Participants were randomly assigned to one of the four conditions. They received a similar questionnaire as in Study 1, with the differences outlined below.

*Control manipulation*. This manipulation differed slightly from that used in Study 1. Participants read a statement about the impact of the economic crisis on people’s lives (people are able to cope with and control the effects of the crisis in their lives / people are not able to cope with and control the effects of the crisis in their lives) [2], and were asked to write down two controllable or uncontrollable effects (high control vs. low control) that the economic crisis had on their life (see Appendix 1 in S1 File).

#### Measures

*Perceived control*. The same 3 items were used as in Study 1. Still, the reliability of the scale remained low (*α* = .54) and the items were considered separately.

*General voting intentions*. This scale evaluated the intentions of voting on a scale from 0 ("I would not vote again in the next elections") to 100 ("I am completely sure that I will vote in the following elections”).

*Support for the parties*. The same 3 items as in Study 1 were used. The reliability was good for all the parties (*α* = .92 to .96) except for the socialist party (main party in the opposition, *α* = .42).

*Support for a change in the voting system*. Support for a change in the voting system was measured with two items: *To what extent do you agree with a reform of the Electoral System so that there is a greater possible proportionality between the number of votes and the number of parliamentary seats*?*/Would you agree with a single constituency model in which the actual percentage of votes was proportionally reflected in the number of seats*? Participants rated their willingness to change the electoral system on a 7-point-scale (ranging from 1 = not at all to 7 = totally, *α* = .76).

### Results

We conducted a Univariate ANCOVA with Control (high vs. low) as a factor and Political Efficacy as a covariate on each of the dependent variables and a MANCOVA on the support for the different parties. Results did not differ if the covariate was excluded.

#### Perceived control

As in Study 1, there was no effect of the control manipulation on the perceived general control items. All, *F*s < 1, ns.

#### Support for the parties, general voting intention and support for a change of the voting system

**Fig 2:**
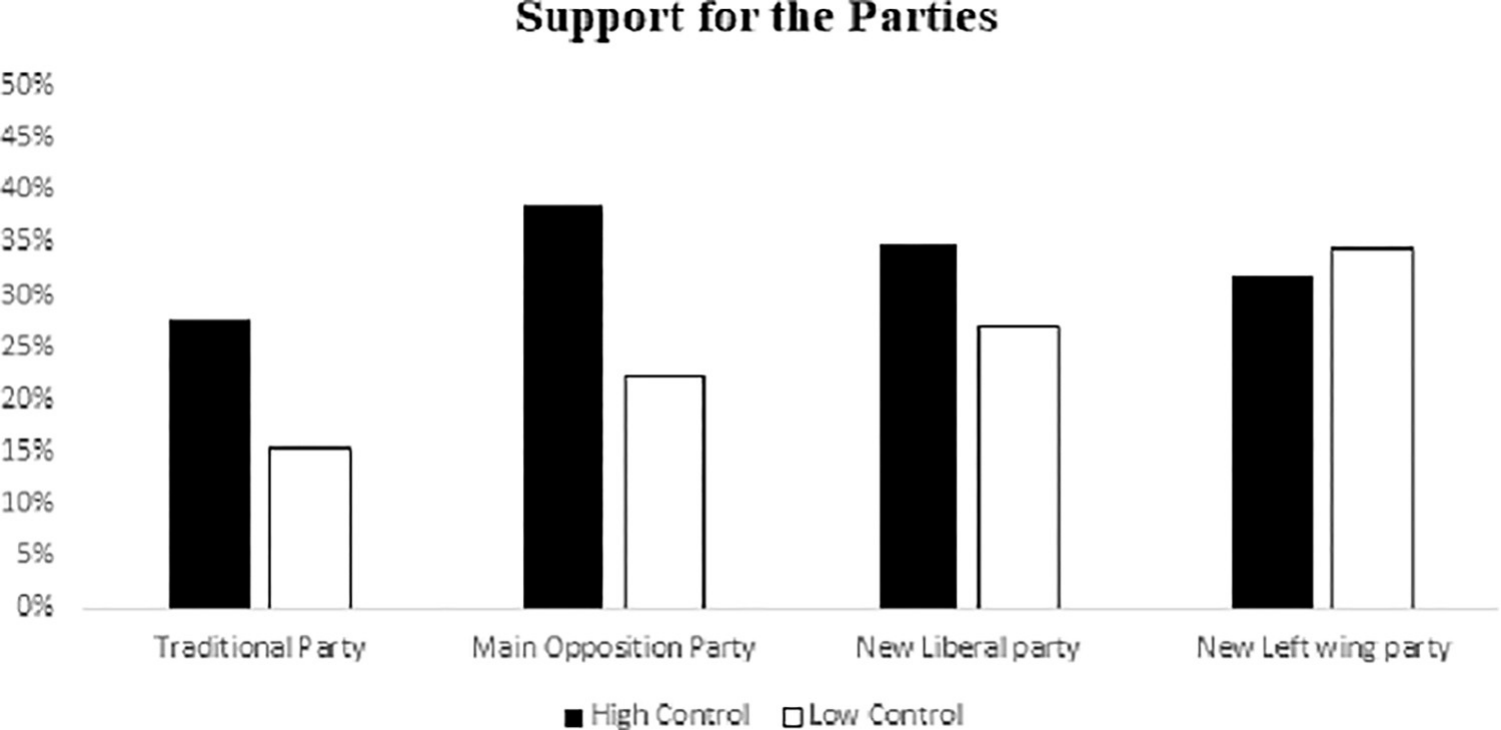
Support for the different political parties by experimental control condition (Traditional party PP).

Before analyzing the different scales of support for the different political parties, we analyzed the general intention to vote in the next elections, and we did not find any effects of the manipulated variables, *F*<1, ns. Regarding support for the specific parties, we expected to replicate the result of Study 1 such that low control would predict reduced support for the traditional party (Hypothesis 1). We found a significant effect of threat to personal control on support for the traditional party, *F*(1,87) = 4.65, *p* = .034, *η*^*2*^ = .053, and a similar, albeit non-significant trend for the main opposition party, *F*(1,87) = 2.89, *p* = .093. *η*^*2*^ = .034. The governing traditional party was supported less in the low control conditions than in the high control one (Fig 2). Lastly, we did not get significant results in the measure *change of the voting system*, *F*<1, ns.

### Discussion

The results confirmed our first hypothesis, such that threats to personal control led to reduced support for the traditional party in comparison with a condition in which high control was primed, in line with the findings of Study 1. In sum, the results of this and the previous study tend to confirm our main hypothesis that threatened personal control can lead to changes in voting norms, reflected in decreased intentions to support for at least one traditional party. Still, the effects are weak, could be partly explained to both high and low control conditions, and it is unclear whether the manipulation of control refers to perceived control at the personal or the collective level, because it referred to the global economic crisis. This might explain why we do not find significant effects on the manipulation checks of the first two studies, because we are manipulating control at the collective level (related to collective agency) but measure the effects at the personal level (related to the self which can trigger defensive mechanisms) [33]. In order to disentangle these two factors, in the next studies we manipulated them orthogonally. According to the group-based control model, ingroup support and defense should be most prominent when both personal and collective control seem at stake [4, 9].

One limitation of the first two studies is that we did not assess the political orientation of the participants. Political orientation can strongly influence their support for the different parties and could partly account for the effects of control on our dependent variables. For this reason, we included this measure in the subsequent studies.

Finally, as in study 1 the manipulation of personal control threat did not influence general feelings of control over one’s life, but it possibly activates the idea that the effects of the economic crisis were (un)controllable and urges persons to search for alternatives as explained in Study 1. We include a measure of feelings of control evoked by the specific situation in the next studies.

## Study 3

Only an agentic group can help to restore control, therefore, threats to personal control could lead to reduced support for traditional parties but only when the ingroup is presented as non-agentic [12]. However, when change is not probable, the opposite pattern might be expected. Thus, in the context of a political impasse, following the group traditional norm (e.g., supporting the system-affirming party) might be the most agentic response to threat to personal control (Hypothesis 1). Alternatively, when control is threatened, but the group is perceived as agentic, arguably there is more hope and scope for change and thus, participants might distance themselves from normative voting tendencies by supporting the traditional parties less (Hypothesis 2). Study 3 was conducted in October 2016. At that point in time, Spanish citizens had gone through two rounds of general elections in less than 6 months with no clear outcome that could lead to the formation of a legitimate government. Before the third round of elections, public opinion was clearly in favor of overcoming the blockage [34] and the traditional party in the government was the only one that could reach the sufficient majority to overcome the impasse. Therefore, there was a reality constraint that impeded perceptions that actual change was possible. According to social identity theory, when there is little scope and hope for social change, people are more likely to accept the system as legitimate [11]. We conceptualize the ingroup at the national level (Spanish people) as we are measuring the effects of personal control threats on voting intentions in the Spanish general elections.

### Materials and methods

#### Participants and design

The study was carried out on a total sample of 143 Spanish nationals from the general population, 67 of whom were women and 76 men, with an average age of 32.28 (*SD* = 15.02). Following the same criteria of exclusions as in the previous studies, 4 participants who did not fill in the manipulation task or had missing values on the focal variables were excluded. This study followed a 2x2 between-group design (Control salience [high vs. low] x Group agency [high vs. low]). We conducted a sensitivity analysis using G*power [28]. Results showed that with this sample size (*N* = 141), the minimum effect size that we can detect for an ANOVA 2 x 2 is *α* =. 05 and 1-*β* = .80 is *f* = .22 (minimum detectable effect). Data collection was carried out by means of an anonymous questionnaire distributed at a bus station and on campus, which helped us to obtain a more representative community sample (65 participants were students and the rest of the sample was formed by community members).

The collection period ran from 14 to 17 October 2016, when the main political parties in Spain were still negotiating in order to form a government and there was no certainty about whether the elections would have to be repeated a third time.

#### Procedure and measures

After signing the consent form, participants received the materials described below. Participants were randomly assigned to one of the four conditions.

*Political orientation*. We measured political orientation on a scale from 0 (extremely left-wing) to 100 (extremely right-wing).

*Control manipulation*. To make sure that the effects described in studies 1–2 are not just specific to the threat to personal control assessed in the context of the economic crisis, we used a manipulation task that relates to general aspects of personal control. Instead of mentioning economic recession, we asked participants to think of and describe one situation of their lives that they can (or cannot) control [9]. We assigned participants to one of two conditions—high vs. low personal control.

*Perceived control*. We modified this measure to avoid referring to general feelings of control over one’s life, and focused instead on the experience of control evoked by the specific situation. After completing the manipulation participants were asked to show on a 10-point scale (0 = none control, 10 = total control) how much they felt in control in that moment (*To what extent did the situation you described make you feel you had control over what was happening*?).

*Group agency manipulation*. We created two texts recreating a historical fact about the independence war between France and Spain at the beginning of the 19^th^ century, in which we activated either low or high agency of the ingroup (Spanish). We used the definition of group agency in terms of group free consensus about common goals, the coordinated actions of the group towards those goals and the ability of the group to attain goals [23]. In the low agency condition the inability of the Spanish people to prevent the coronation of a French king was emphasized, whereas in the high agency condition the effectiveness of the Spanish people to depose the illegitimate king was activated (see Appendix 1 in S1 File).

*Perceived group agency*. To check if the group agency manipulation worked properly, we asked “What degree of group agency of the Spanish people did the example you have been presented with describe?”. Participants answered on a scale from 0 (Low group agency) to 10 (High group agency).

*General voting intentions*. As in Study 2, participants were asked to indicate how likely it was that they would take part in the next elections on a scale from 0 to 100.

*Support for political parties*. Participants were asked about their support for the four main parties included in the polls (two traditional parties and two new parties) with the same 3 items as in the previous studies on a scale from 0 to100. The reliability for this index was between *α* = .85 and *α* = .94.

*National identity*. We used 3 items taken from Leach et al.’s (2008) [35] identification centrality scale and one general item [36]. Participants answered on a seven-point scale (ranging from 1 –Totally disagree to 7 –Totally agree): *I identify with the Spanish as a group/I have strong ties with the Spaniards/In general*, *being Spanish is an important part of how I see myself /Being Spanish is important to me*. (*α* = .89).

*Group efficacy*. In order to measure the efficacy of the Spaniards as a group, we used 3 items adapted from van Zomeren et al. [37] (2008) on a 7-point scale (from 1 –Totally disagree to 7 –Totally agree). The items were as follows: *I think the united Spaniards can successfully defend their rights/I think the united Spaniards can successfully overcome their difficulties/I think the united Spaniards can improve their status in society* (*α* = .94).

### Results

#### Perceived control and group agency

There was a significant main effect of the control manipulation on perceived control, *F*(1,138) = 498.08, *p <* 0.01, *η*^*2*^ *=* 0.79. Participants in low control conditions perceived less control (*M* = 1.47) than participants in high control conditions (*M* = 8.47).

Finally, there was a significant effect of the agency manipulation on the agency manipulation check, *F*(1,138) = 132,82, *p <* 0.01, *η*^*2*^ *=* 0.49. In the low agency condition, participants perceived less group agency (*M* = 2.58) than in the high agency condition (*M* = 7.16).

#### General voting intention

There was no effect of threat to personal control manipulation on general voting intentions, *F*(1,139) = 3.16 *p* = .074 *η*^*2*^ *=* .02. The pattern suggests that in low control conditions, the probability of participating again in the next elections tended to be higher (*M* = 77.63) than in high control conditions (*M* = 66.80). This effect was not moderated by agency, *F* (1,139) = .78, *p* = .378, *η*^*2*^ < .01.

#### Support for political parties

**Fig 3:**
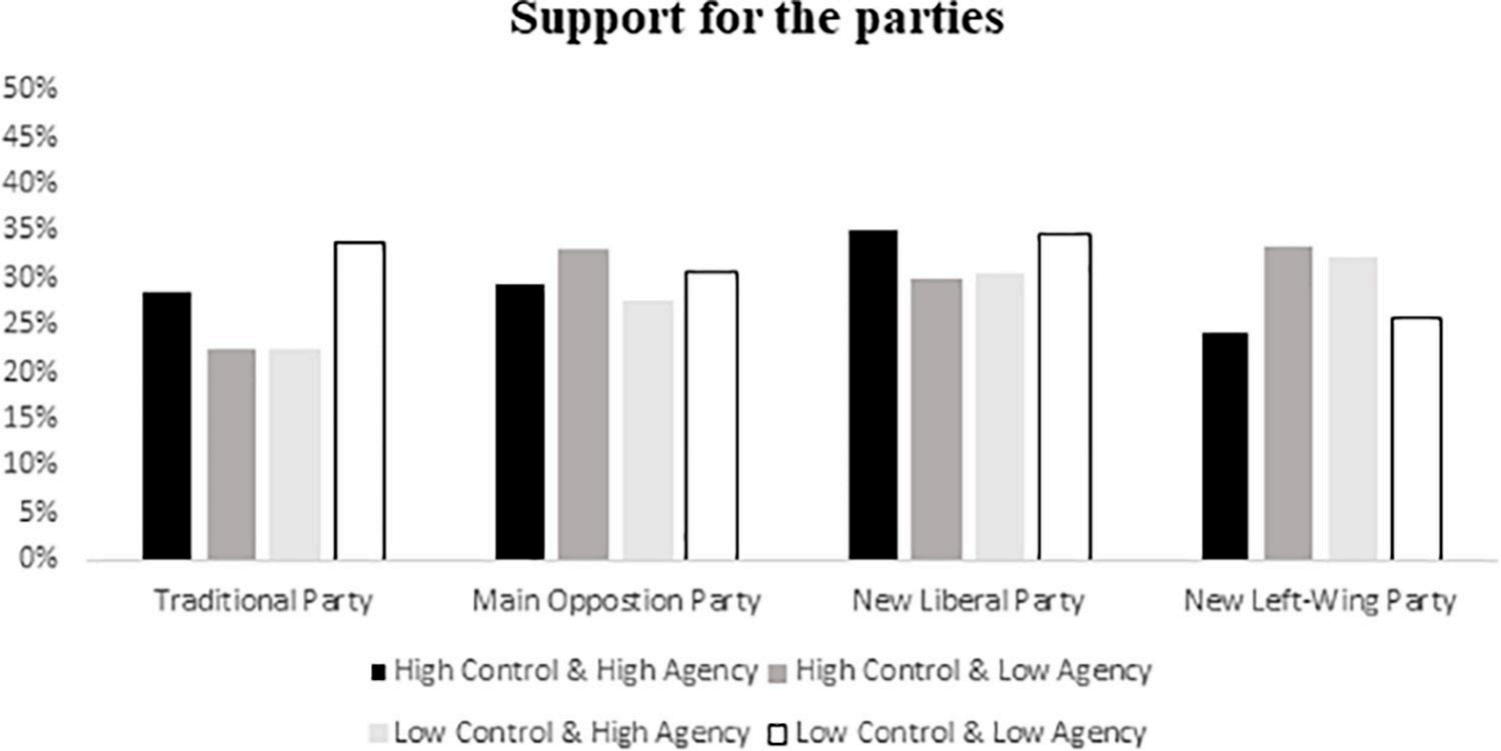
Support for the different political parties by experimental conditions.

In order to test our hypotheses, we conducted a 2 x 2 MANCOVA including control and agency as independent variables and support for the different parties as dependent variables. We also included political orientation as a covariate. We expected to find that participants in low control conditions would show more support for traditional parties when group agency was low (H1) and reduce it when group agency was high (H2). The results of the analysis showed no multivariate significant effects, all *F*s ≤1.93, *ns*, all *p*s ≥.38. Univariate analyses showed a significant interaction of control and agency on support for the traditional party in the government, *F*(1,140) = 4.61, *p* = .034, *η*^*2*^ = .03. Planned comparisons indicated that when group agency was low, participants supported the system-affirming party more in the low control (*M* = 28.87) than in the high control condition (*M* = 20.41, *F* (1,140) = 4.61, *p* = .034, *η*^*2*^ = .033). However, under conditions of high agency, support for the government tended to be lower in low control (*M* = 19.38) compared to high control conditions (*M* = 24.57; *F*(1,140) = 3.58, *p* = .060, *η*^*2*^ = .03; Fig 3). Results did not vary significantly when political orientation was not included as a covariate. Descriptive and correlations appear in Table 1. Further information about mean scores and standard deviations for support for the different political parties by experimental condition can be found in S1 Table in Appendix 2 in S1 File.

**Table 1:** Means, standard deviations and correlations for support for the parties and national identity.

*DV’S*	*M*	*SD*	*1*	*2*	*3*	*4*	*5*
1.Support for traditional party	23.28	29.37	-	-.06	-.46**	.60**	.41**
2. Support for main opposition party	30.40	24.72		-	.09	.12	.26**
3. Support for new liberal party	32.57	32.47			-	-.31**	-.34**
4. Support for new left-wing party	30.33	28.44				-	.36**
5. National Identity	4.54	1.62					-

* indicates *p* < .05.

** indicates *p <* .01.

#### Group efficacy and national identity

For the group efficacy variable, we obtained a significant effect of collective agency in a MANCOVA using political orientation as a covariate, *F* = 7.75, *p* = .006, *η*^*2*^ *=* .05: Those who were made aware of low collective agency reported less group efficacy (*M* = 5.36) compared to those in the high collective agency condition (*M* = 5.98). Finally, the control variable and the interaction between personal control and agency did not report significant results, all *F*s ≤ .99, *ns*., all *p*s ≥ .05, *ns*.

For the national identity variable, we did not obtain any significant results over agency, *F* ≤.46, *p* ≥.21, *ns*., and for the control variable the results are also not significant, *F*≤.21, *p* ≥.46, *ns* which was also the case for the interaction between both variables. See Table 1 for descriptive and correlations.

### Discussion

The results showed that participants in the low control condition (vs. high control) supported the traditional party *more* when group agency was low. Thus, threats to personal control can lead to support for normative parties when group agency is low. This result is partly contradictory to our previous findings, although we had not manipulated collective agency previously. This pattern of results might be because, at the time the study was conducted, Spain faced a political impasse, which seemed to be leading to a deadlock when no government had been formed for over 7 months. To determine whether our hypotheses regarding the effect of threat to personal control on changes in normative voting patterns would be supported in a context in which change could actually take place, we conducted the next study in a different national context (France) in which political change could happen.

## Study 4

We generally hypothesize that lack of control should influence support for traditional parties when group agency is low depending on whether change or stability are perceived as the most strategically agentic responses. Study 3 provided support for this prediction in the context of a political impasse, where following the ingroup norm through supporting the traditional party in the government was seen as the only way out. But do people with low personal control *reduce* their support for traditional parties in a context in which change is possible? In Study 4, we tested this hypothesis in France, just before the legislative elections and shortly after presidential elections in 2017. Until that date, the French Parliament was mainly composed of deputies from two parties: the ruling *Parti Socialiste* (left-wing), and the main opposition party–*Les Republicains* (right-wing). However, the situation had utterly changed in 2017, when new parties (such as *En Marche* or *La France Insoumise*) and an old system-affirming party, previously rather unpopular (*Front National*), gained stronger support. Therefore, at the time the study was conducted, France was in the middle of a political change. The data were collected right after the presidential elections in which, for the first time, the candidates from the two traditional parties had failed to go to the second round of election. Therefore, the context of change was highly salient in the country at that moment. Because our main hypothesis referred to the effect of low control on support for traditional parties when group agency was perceived as *low*, we manipulated only control salience and kept agency constantly low between participants. We predicted that participants in the low control condition will be less likely to support the traditional parties compared to those in the high control condition.

### Materials and methods

#### Participants and design

Participants were 82 French nationals with voting rights in French legislative elections, who took part in 2017. After excluding 7 participants who refused to fill out the manipulation task, the final sample consisted of 75 participants (53 Woman, *M* = 30.21, *SD* = 11.45). A sensitivity analysis showed that with this sample size, the minimum effect size that we can detect for *α* =. 05 and 1-*β* = .80 is *f* = .33 (minimum detectable effect). The study was conducted online and the data collection started two weeks before the elections (in May 2017). The study followed a one factorial design (high personal control vs. low personal control). It was conducted using SurveyMonkey and, as in Study 1, distributed via different social media platforms.

#### Procedure and measures

We administrated to participants the following materials:

*Control manipulation*. We used a similar manipulation as in Study 3 with the difference that participants had to think about and describe two aspects of their lives that they could (or could not) currently control instead of one situation [9]. Participants were randomly assigned to one of two conditions–high vs. low personal control.

*Perceived control*. After completing the manipulation task, participants were asked to indicate on a 7-point scale (1 = not at all, 7 = very much) how much they felt in control of their lives at that moment.

*Low collective agency prime*. In Study 4, we aimed to keep group agency on the same level among participants (low agency salient). All participants were presented with the description of a current political situation in France that triggered low collective agency. Specifically, they were reminded of the protests organized in France between March and September 2016 that mobilized hundreds of thousands of people to demonstrate against the new labor law reform presented by the government. However, despite the scale of the protest, the government had not given up on their project, which resulted in failure of one of the most important movements of this type in France in the 21^st^ century. The original text used in the study is available in Appendix 1 in the S1 File.

*Collective agency*. To check whether we managed to keep the level of perceived ingroup agency constant between participants in two conditions (high vs. low personal control) we added 2 items from the collective agency scale developed by Stollberg et al. [12]. These items were “In general, the French have common goals they are able to achieve” and “I think the French are able to achieve common goals”, *r* = .29, *p* = .011. Due to a relatively weak correlation, we analyzed items separately.

*General voting intentions*. Similar to the previous studies, participants were asked to indicate how likely it was that they would take part in the next legislative elections in France on a scale from 0 to 100.

*Support for political parties*. It was measured with the same 3 items as in previous studies. The alpha level for support for each of the parties was between *α* = 0.89 and *α* = 0.93.

*Political orientation–votes in the previous elections*. Participants’ political orientation was measured with a question asking who they voted for in a presidential election that took part in April 2017. We recoded participants’ answers to a scale from 1 to 8 where 1 stood for the most left-wing candidate and 8 stood for the most right-wing candidate (Each candidate chosen by participants was coded as follows: 1 –Philippe Poutou, 2 –Jean-Luc Mélenchon, 3 –Benoît Hamon, 4 –Emmanuel Macron, 5 –François Fillon, 6 –Nicolas Dupont-Aignan, 7 –Jacques Cheminade, 8 –Marine Le Pen. Answers of those participants who did not take part in the elections or who cast a blank vote were treated as missing values). We also asked participants to indicate who they voted for in the second round of the elections. Other measures have also been used and can be consulted in Appendix 2 in the S1 File.

### Results

All dependent variables were analyzed using analysis of variance, including control factor as the main IV and controlling for political orientation as a covariate.

#### Perceived control

We found that participants in the low control condition felt less in control than participants in the high control condition (*M*_1_ = 3.82, *M*_2_ = 4.61, *F*(1,73) = 6.00, *p =* .017, *η*² = .08).

#### Perceived agency

We checked whether control manipulation influenced both items measuring perception of ingroup agency (agency of French people). As expected, there were no significant differences between low and high control participants for the first item (*M*_1_ = 3.86, *M*_2_ = 3.74, *F*(1, 63) = 0.10, *p =* .758, *η*² < .01), or for the second item (*M*_1_ = 4.29, *M*_2_ = 4.26, *F*(1, 63) = 0.03, *p =* .865, *η*² < .01). Therefore, we kept it constant among participants. The mean of these two items (*M* = 4.10) was not statistically different from the midpoint of the scale (4), *t*(74) = 0.73, *p* = 0.470.

#### Support for political parties

**Fig 4:**
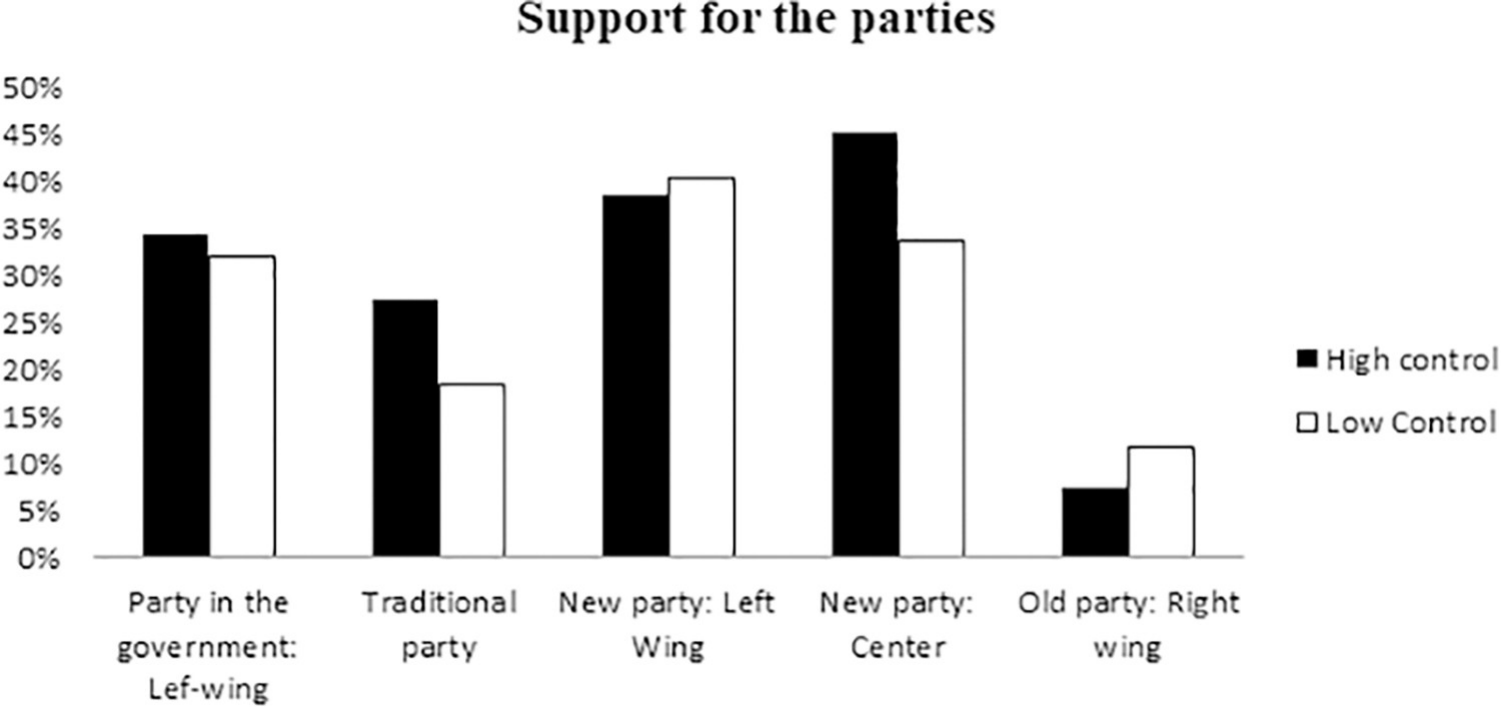
Impact of control manipulation on support towards different parties (Traditional party: Les Republicains).

To test our hypothesis that threat to personal control decreases support for the system-affirming parties, we used a MANCOVA with control as the independent variable and support for the parties as dependent variables. We found that threat to personal control decreased support for the traditional party (Les Republicains) that served as the opposition party (*M*_1_ = 18.48, *M*_2_ = 27.53, *F*(1, 63) = 4.19, *p =* .045, *η*^2^ = 0.06; Fig 4) in comparison with high control. Control manipulation did not affect support for any other party, all *p* > .12. Results without political orientation as a covariate show the same pattern, although the interaction became non-significant, F (1,75) = 2.06, *p =* .156 *η*^2^ = .294.

Measures such as General Voting Intentions, Control Restoration Scale and other results like interaction of control manipulation and perceived efficacy of French people on voting intentions in legislative elections and mean scores and standard deviations (in parentheses) for the main dependent variables by experimental condition can be consulted in Appendix 2 in the S1 File.

### Discussion

We conducted Study 4 at a time when France was in the middle of a major political change; namely it was carried out in the same month as, for the first time in years, a candidate from a new party different from two traditional ones (Emmanuel Macron, leader of *En Marche*!) became the President of the country. Our aim was to test whether, in this change context, the threat to personal control may decrease support for traditional parties. Indeed, we found that in the low control condition, participants were less likely to support the traditional party than those in the high control condition. These results support our hypothesis, although we cannot exclude the possibility that they might be partly driven by high control condition. Contrary to Study 3, which was conducted in Spain when change was seen as improbable, setting a reality constraint for our hypothesized effect of threat to personal control on support for traditional parties, in this study change was perceived as a real possibility, hence even when low group agency was activated across conditions, a change in voting intentions and support towards one of the traditional parties was observed as a function of threat to personal control.

## Study 5

Studies 3 and 4 provided opposing evidence about the impact of threat to personal control on support for traditional parties when group agency is low in two different contexts in which stability or change could be seen as the most agentic responses to deal with threat to personal control at the time the studies were conducted. As previously explained, this incongruence might be due to the situational factors that created a reality constraint in Spain, where change was improbable (a stability strategy was preferred), versus a highly changing political situation in France (political change was a real possibility). In order to provide empirical support for this situational explanation, in Study 5 we experimentally manipulated stability versus change under low agency conditions. We tested the effect of threat to personal control on support for the parties when group agency was constantly low and stability versus change were primed as the best (e.g., most efficient) political strategies in times of crises. Based on the findings of Study 4 we hypothesized that threat to personal control would produce reduced support for traditional parties when change is emphasized (Hypothesis 1), and also based on the findings of Study 3 we hypothesized that support for traditional parties would be increased when stability is primed (Hypothesis 2).

### Materials and methods

#### Participants and design

The study was carried out with a total sample of 255 Spanish nationals, 164 of which were women and 91 men with an average age of 24.49 years (*SD* = 9.04). 47 participants had to be excluded according to the same exclusion criteria as in the previous studies, because they did not answer the manipulation task or had missing values on the focal variables. We conducted a sensitivity analysis using G*power [28]. Results showed that with this sample size (*N* = 255), the minimum effect size that we can detect for *α* = .05 and 1-*β* = .80 is *f* = .40 (minimum detectable effect).

The data collection was conducted using an anonymous paper-based questionnaire at a bus station (community sample). The collection period occurred between 13 and 30 June 2017. The political situation in Spain at that time was that a government was formed by the traditional party after two failed elections.

#### Procedure

At the beginning we measured the political orientation of participants, and then asked them to imagine and write down two situations in which they had experienced full control or lack of it (depending on the experimental condition). Afterwards, they read a brief text emphasizing the need for stability vs. change to overcome the crisis. Thus, we randomly assigned participants to one of the four experimental conditions.

*Control manipulation*. We used the same manipulation task as in Study 3, in which participants had to think of and describe one situation in their lives that they could (or could not) control.

#### Measures

The materials were presented in the following order.

*Political orientation*. We measured political orientation on a scale from 0 (extremely left-wing) to 100 (extremely right-wing).

*Perceived control*. We measured perceived control with the same item as in Study 3.

*Low agency priming*. We used the same text as in Study 3 to activate low group agency. Note that in this study agency was not manipulated, but it was held constantly low across conditions.

*Perceived group agency*. We measured perceived group agency with the same item as in Study 3.

*Political strategy manipulation (Change vs*. *stability)*. We created two scenarios presenting experts’ perspective regarding what is best for a country to maintain (vs. change) government in the case of a crisis (see Appendix 1 in S1 File).

*Political strategy manipulation check*. Here we asked participants: *To what extent do experts consider that a change of government would be positive for Spain*? This scale ranged from 0 to 10, where 0 stood for “Not positive” and 10 for “Very positive”. This question was created to test if our manipulation worked as expected.

*General voting intentions*. As in the previous studies, we asked participants how likely it was that they would take part in the next legislative elections if there was another round of legislative elections in Spain on a scale from 0 to 100.

*Support for the parties*. The same three items as in the previous studies measuring closeness, voting intentions, and perceived efficacy of each party were used. The reliability for this index was between *α* = .90 and *α* = .95.

*National identity*. We used the same scale as in Study 3 (*α* = .91).

*Group efficacy*. As in study 3 & 4 we used the same items (*α* = .91).

### Results

#### Perceived control and perceived political strategy

A 2 x 2 MANOVA was carried out for perceived control, agency, and political strategy as dependent variables. There was a significant effect of the control manipulation on perceived control, *F*(1,205) = 676.9, *p* < .001, *η*^2^ = .77, such that in the situation of low control, the participants perceived themselves as having felt less control in that situation (*M* = 1.59) than in the situation of high control (*M* = 8.04). There was also a significant main effect of the political strategy manipulation on the corresponding manipulation check, *F*(1,206) = 50.76, *p* < .001, *η*^2^ = .20. We can observe that when stability is emphasized there is less support for accepting political change (*M* = 5.70). However, when change is primed there is higher support for accepting political change (*M* = 7.94).

#### Voting intention and support for the parties

First, we carried out a 2 x 2 ANOVA on voting intention. The main effect of stability versus change manipulation was not significant, *F*s<1, ns. There were no significant effects of control or interaction on this variable, *F*s<1, ns. Descriptive statistics and correlations appear on Table 2.

**Table 2:** Mean, standard deviations and correlations for support to the parties and national identity.

*DV’S*	*M*	*SD*	*1*	*2*	*3*	*4*	*5*
1.Support for traditional party	26,37	30.80	-	-.089	.54**	-.44**	.42**
2. Support for main opposition party	28,99	24.63		-	.16*	.14*	.03
3. Support for new liberal party	32,97	27.96			-	-.41**	.41**
4. Support for new left wing party	29,41	32.96				-	-39**
5. National Identity	4,56	1.78					-

* *p* < .05.

** *p <* .01.

**Fig 5:**
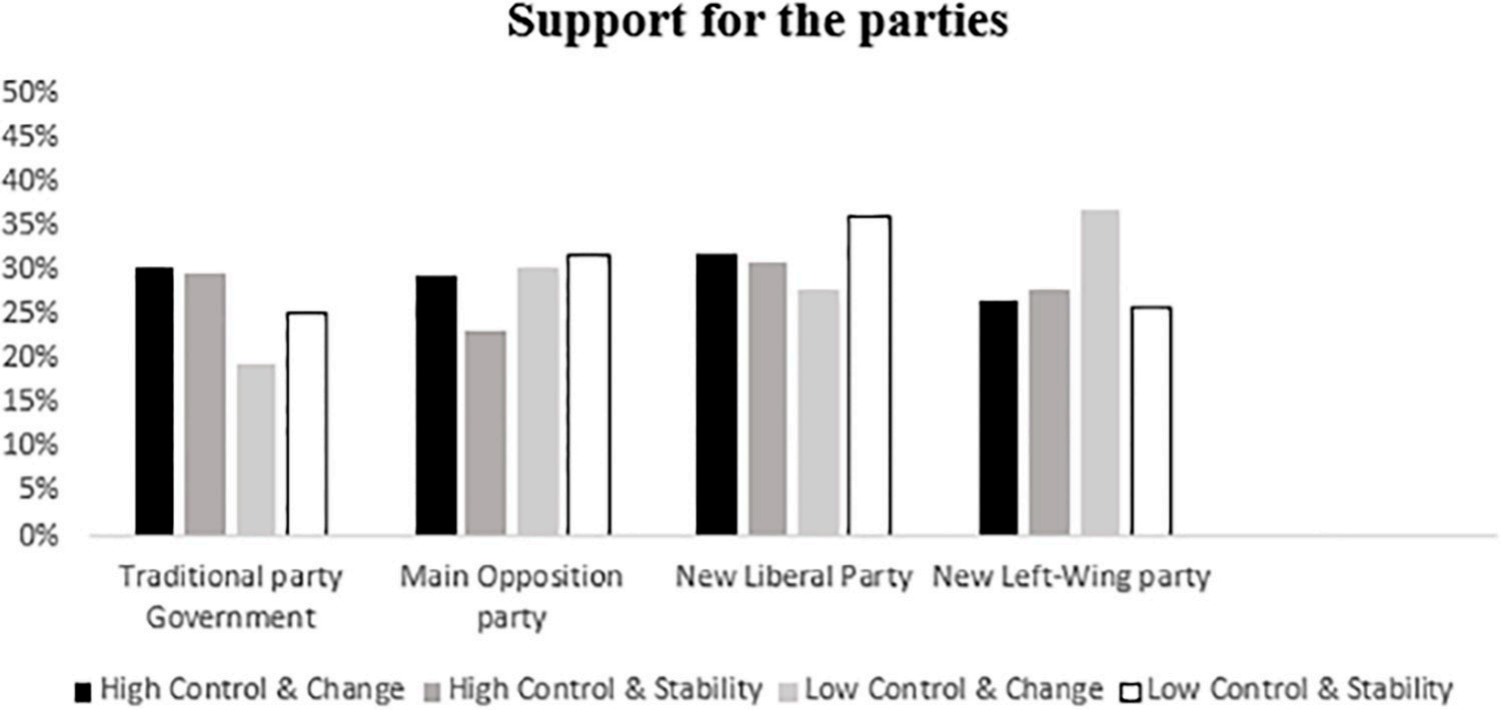
Support for the different political parties by experimental conditions (traditional party: PP).

We conducted a 2 x 2 MANCOVA to test the main hypotheses of this study, using the manipulation of control (high vs. low) and the best strategy of acting (change vs. stability) as fixed factors, political orientation as a covariate and support for the different parties as dependent variables. There were no significant multivariate effects, all *p*s > .05. The analysis of univariate effects showed a main effect of threat to personal control on support for the traditional party in the government, *F*(1, 204) = 5.66, *p* = .018, *η*^*2*^ = .028, indicating that participants in the low control conditions supported the system-affirming party less (*M* = 23.38) than in high control ones (*M* = 30.18). This effect was not significantly moderated by political strategy (stability vs. change), *F*(1,204) = .615, *p* = .434, *η*^*2*^ < .01. However, the planned comparisons showed that the effect was only significant for the condition in which change was promoted as the best political strategy (Fig 5), *F*(1, 204) = 5.06, *p* = .026, *η*^*2*^ = .025, whereas no significant effect of threat to personal control appeared in the stability condition, *F*(1, 204) = 1.26, *p =* .263, *η*^*2*^ < .01 (see S5 Table in Appendix 2 in S1 File). Results did not vary significantly if political orientation was not included as a covariate.

#### National identity and group efficacy

**Fig 6:**
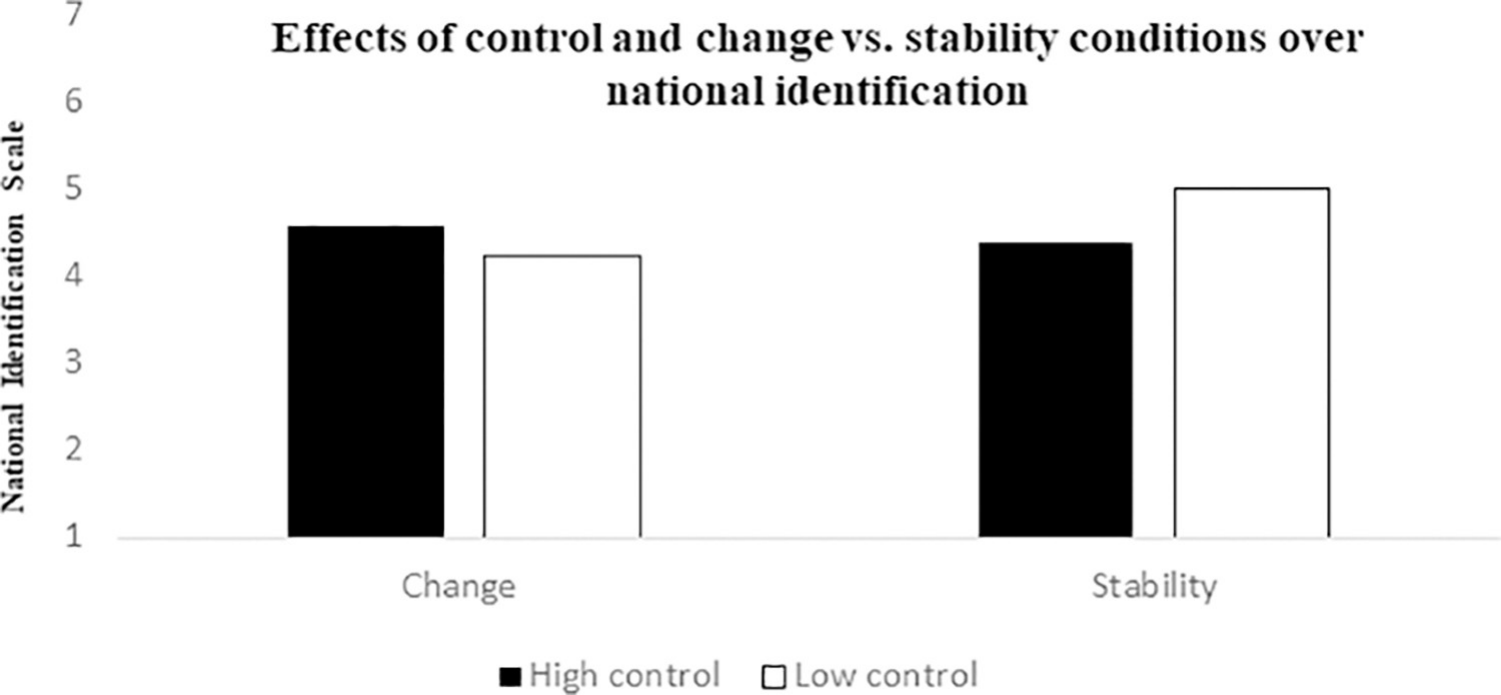
Effects of control and change vs. stability conditions on national identification.

We also found a significant interaction of political strategy (change vs. stability) and participants’ level of national identification using political orientation as a covariate, *F*(1, 204) = 4.93, *p* = .027, *η*^*2*^ = .02. Pairwise comparisons showed that in the low control conditions there was a significant effect of political strategy on national identification (Fig 6), *F*(1,204) = 4.81, *p* = .029, *η*^*2*^ = .02; those who perceived low control and when stability was primed as the best political strategy felt more strongly identified with their national ingroup (*M* = 4.97) than when change was primed as the best political strategy (*M* = 4.33). There was no effect of strategy priming in the high control conditions, *F*(1,204) < 1, *p* = .330, *η*^*2*^ < .01. The interaction effect became non-significant if political orientation was not included as a covariate, *F*(1,206) = 3,81, *p* = .052, *η*^*2*^ = .01, but the pattern of results was the same, and paired comparisons remained significant, *F*(1, 206) = 4,83, *p* = .029, *η*^*2*^ = .023.

Considering the group efficacy variable, we did not find any significant results, *F ≤* 3.12, *ns*, *p* > .05, *ns* (see Table 2 for descriptive statistics and correlations).

### Discussion

Overall, in Study 5, threat to personal control led to reduced support for the traditional party in comparison to a high control condition. This replicates the findings of Studies 1, 2, and 4. As in previous studies, we need to be cautious when interpreting this finding exclusively according to our predictions, as priming high control could have partly contributed to the effects. Regarding the role of stability versus change, the results are not conclusive, although the a priori planned comparisons are in line with our predictions that the effect should occur primarily when change is emphasized. Still, the condition in which the traditional party received the least support was the one in which personal control was threatened and change was seen as the most effective coping strategy, which is in line with the findings of Study 4 in France, where political change had already taken place.

Of interest, low control led to higher ingroup identification when stability was primed as the best political option. This result is consistent with the group-based control restoration literature that has found increased ingroup identification under conditions of threat to personal control, particularly when the group was perceived as agentic [12], or stable [38]. Specifically, in the stability condition of the present study, participants were likely to infer that the current shape of the national ingroup promises collective agency.

Correlational analyses showed that national identification is positively correlated with voting intentions and support for the traditional party and negatively correlated with the new left-wing party that challenges the system. It is possible that, in the stability condition, threat to personal control leads to increased national identification and higher support for traditional parties. These effects could explain the findings of Study 3 in which threat to personal control and group agency led to the highest support for the traditional party when change was not seen as possible.

## General discussion

Across four out of five experimental studies, we showed preliminary evidence that loss of personal control can evoke a willingness to change non-agentic political systems of the ingroup. When the context of an economic or political crisis was salient, people whose sense of personal control was threatened were less likely to support traditional parties that represented the old system [2]. Such results, supporting our hypotheses, occurred in Studies 1, 2, 4 and 5. Although we did not find an effect of the threat to control manipulation on general feelings of control over life in our first two studies, one might still argue that such a manipulation triggers feelings of low controllability of the economic effects of the crisis in line with previous research [2]. In Study 3 we found the *strongest* support for the traditional party when both personal control and group agency were low. This result is the only one that aligns with compensatory control theory [20], and system justification theory [39] suggesting that people might accept the system as legitimate, or promoting a conservative shift when they perceive the group as unable to carry out social change [21]. This was the case in Spain in the context of a political impasse, in which Study 3 was conducted (after two rounds of political elections in 6 months and the failure of political parties to form a functional government). On the contrary, when change was highly salient and possible (Study 4, after a presidential change in France; Study 5, experimentally manipulated in Spain), and collective agency was low, threats to personal control decreased support for the traditional party. Thus, our hypothesis about reduced support for traditional parties when control is threatened was conditionally confirmed in our studies, but only when political change was possible or pictured as the most efficient solution. This supports an integrated model of group-based and compensatory control [8, 21]. People respond to threatened personal control with group-based control restoration (“extended primary control”; Fritsche, 2022) as long as a relevant in-group is cognitively available that people consider potentially agentic or for which establishing group agency seems possible. If such a group is not available in a given situation, people are expected to turn to establishing order and structure in their environment (“secondary control”) through affirming existing social systems, as predicted by compensatory control theory [40]. For people, this reduction of uncertainty may lay the ground for restoring personal control at a later point in time.

### Threat to personal control and perceived group agency lead to a change in support for the parties

There are different “routes” to restore threatened personal control: personal strategies and collective coping strategies [21]. One way to restore control is to change the environment in which people live and voting entails the most efficient way to achieve such a change in democratic societies [41, 42]. In this research, we focused on voting behavior as a restorative tool that allows a person to maintain their own sense of control via her or his collective self [4, 13]. The specific socio-political context determines whether support for maintaining a stable system or changing it is perceived as a more agentic form of coping with personal control threats. It also sets up the limits of controllability, that is, imposed reality constraints often make change impossible [43]. This is consistent with social identity theory, which predicts that social change is unlikely to occur when stability is emphasized [11], and with system justification theory, which claims that individual or group threats will lead to validating the establishment [39, 44]. However, when the situation is perceived as unstable or illegitimate, actions towards social change will emerge [11]. In such unstable conditions, when normative behaviour no longer serves its function to protect the ingroup, individuals might react by distancing themselves from the norm or by searching for alternatives [45]. Our research findings suggest under what type of conditions threatened personal and collective control can lead to support for political change. Two factors seem to be crucial to determine such boundary conditions–agency of the political party and the functionality of the coping strategies of focusing either on change or stability (including a possible scope for change). Further research should focus more on those contextual factors that might play an important role in understanding when threats to personal control lead to support for traditional vs. new political parties.

The present results can contribute to better understanding how and why personal and collective control threats interact. Previous research on group-based control has repeatedly found that people engage in group-based action most vigorously when lacking personal control and threat to collective agency are salient at the same time [4, 9]. We found evidence of this pattern in two of our studies (Studies 4 and 5). However, we also identified a crucial boundary condition of this effect in Study 3, suggesting that this pattern does not occur when there is a situation of political impasse. That is, people only seem to search for change in response to a lack of personal control when collective agency is low if the current situation allows for the restoration of collective control (Studies 4 and 5) and their specific group-based actions are appropriate means to restore collective agency (Study 5). Future studies should test this in different and more controlled settings.

The results suggest that pursuing collective change might be a viable means of group-based control. In fact, groups (e.g., nations) that pursue change might signal that they are acting due to intrinsic and distinct collective goals, which should stress their agency. Also, this indicates that following threat to personal control, at least some people under certain circumstances do not necessarily become more conservative as proposed in the literature on conservative shift and compensatory control [19, 41]. Instead, our research opens the possibility that some people may prefer collective efforts of control restoration over reducing uncertainty seeking for structure [13]. This is consistent with prior research which shows higher social connectedness [46] and prosocial intentions [47] under situations of economic threat due to the 2008 economic recession.

### Limitations and future research

We are aware of some limitations of this research. First, our studies were not preregistered, which renders our predictions exploratory. Further, most of the sample sizes are small and therefore the statistical power was often insufficient to detect significant effects. The conditions under which the studies were run and the limited time to recruit the samples before the elections constrained the sample sizes. Finally, we only included a baseline condition in the first study, thus we cannot exclude the possibility that our effects may be partly driven by the increase in control in the high control condition. Nevertheless, the pattern of results replicates across 4 out of 5 studies and is consistent with our main hypothesis, supporting our conclusions.

Also, in the first two studies, the manipulation of control did not have a significant effect on items that measured personal control. This is probably due to the manipulation being context specific (related to the consequences of the economic crisis), whereas the items we applied measured a more general sense of personal control. Thus, it was not an adequate manipulation check measure. Previous literature suggests that when people’s sense of personal control is threatened, they attempt to compensate for this threat by expressing higher feelings of control as a defense mechanism [33]. Thus, proximal defense reactions to personal control threat often lead to a denial of threat. This also seemed to be the case in the first two studies reported here, in which personal control threat related to the economic crisis did not impact broader perceptions of control. In the following studies (3 to 5), however, control was not manipulated in the specific context of the crisis and the items measuring momentary feelings of personal control were properly adjusted to the type of manipulation used, resulting in a more viable manipulation check.

We also acknowledge that our samples are primarily female and young people, who have been identified as common actors of social change in the literature. Thus, our findings cannot be generalized to other samples without further evidence [48].

## Conclusions

Our research shows that threat to personal control affects support for political parties. In four out of five studies, we observed that low control led to lower support for system-affirming parties when national agency was at stake because of economical and societal crises. This occurred in different political contexts (regional and national elections and in two different countries), and especially when change was perceived as both possible and effective. However, when low control was accompanied by a sense of low group agency and stability in the sociopolitical context was perceived as the best political strategy, system-affirming parties were *supported*. These findings bolster the notion that rejecting the stability of the national political system, and thus indirectly supporting collective change, can be considered as a means to maintain a sense of control through the collective self. This might be an adaptive response to overcome societal crises.

## Supporting information

S1 File (DOCX)
