# Supplementary material for: Political change as group-based control: Threat to personal control reduces the support for traditional political parties
Source: PLoS One. 2022 Dec 8;17(12):e0278743. doi: 10.1371/journal.pone.0278743 (PMC9731459; doi:10.1371/journal.pone.0278743)
Supplement: S1 File — (DOCX) [file pone.0278743.s001.docx]

**Supplementary Materials**

**Appendix 1**

**Manipulations**

**Study 1**

**High Control Condition.** As a prestigious international economic newspaper states, the measures developed to alleviate the negative effects of the crisis into which we are currently plunged are proving useful, and their impact on the halting the crisis is remarkable. Experts emphasize that the development of the crisis is controllable and will be reduced in the coming months. It is possible to predict how long it will last and how it will evolve. The negative impact on citizens, who are finding ways to face and control the effects of the crisis on their present or medium-term lives, will therefore be reduced. Write a list of 2 controllable effects that the crisis has for your life and for your future.

**Low Control Condition**. As a prestigious international economic newspaper states, all the measures developed to alleviate the negative effects of the crisis into which we are currently plunged are useless, and their impact on curbing the crisis is minimal. Experts emphasize that the development of the crisis is uncontrollable and will continue to unfold in the coming months. It is difficult to predict how long it will last and how it will evolve. It will therefore continue to have a negative impact on citizens, who have no way of coping with and controlling the effects of the crisis on their present or medium-term lives.

Write a list of 2 uncontrollable effects that the crisis has on your life and your future.

**Neutral Condition**. Health professionals affirm the importance of physical activity and sport to maintain a good quality of life, various studies show that despite this in Spain there is not enough sport activities, 4 out of 10 citizens practice sport and just 7 out of 10 walk daily, seeing these data one could say that we are becoming a sedentary society.

Write 2 situations in which you practice sport in your daily life.

**Study 2**

**High Political Efficacy.** Apart from the personal level, there are also measures that can be taken to deal with the effects of the crisis at the political level. Political decisions are made by our democratically elected representatives. At present, the political situation in Spain is quite ambiguous, and it is a key moment in which our representatives can reach agreements on common objectives that allow them to take decisions and carry out proposals that promote relevant changes in Spanish society.

**Low Political Efficacy.** Apart from the personal level, there are also measures that can be taken to deal with the effects of the crisis at the political level. Political decisions are made by our democratically elected representatives. At present, the political situation in Spain is quite ambiguous, and it is not a key moment in which our representatives can not reach agreements on common objectives that allow them to take decisions and carry out proposals that promote relevant changes in Spanish society.

**Study 3**

**High Agency Condition.** Between 1808 and 1814 Spain experienced a period of violence and political instability due to the Napoleonic wars where the Spanish people were invaded by the French Empire. In May 1813, the Spanish people succeeded in deposing Jose I (King imposed by France) from the throne, thus initiating the final stretch towards independence. This is a historical example. Think and write another more recent event in which Spaniards HAVE ACHIEVED to reach a common goal as a people and therefore, have taken the reins of their own destiny.

**Low Agency Conditions.** Between 1808 and 1814 Spain experienced a period of violence and political instability due to the Napoleonic wars where the Spanish people were invaded by the French Empire. In 1808 Joseph I (King imposed by the French) was consolidated on the throne of Spain, making the Spanish people succumb to French interests despite efforts to prevent it. This is a historical example. Think and write another more recent event in which the Spaniards HAVE NOT ACHIEVED to reach a common goal as a people and the destiny of the Spaniards has been determined by external factors or luck.

***Study 4***

**Low agency prime.** In February 2016, the government of Manuel Valls presented the project of the labor law reform, known as the El-Khomeri law. According to the Government, this project was aimed at easing and simplifying the labor law. However, it provoked the unprecedented protest of the French, who regarded it as an infringement of the rights of employees and trade unions, for the benefit of the companies. Between March and September 2016, numerous national demonstrations were organized, mobilizing hundreds of thousands of people of different political orientations protesting against the reform throughout the country. Despite the scale of the protest movement, the government has not given up on its project, introducing in it only a few minor changes. Lastly, lack of a sufficient parliamentary majority, the Prime Minister decided to use an article 49 paragraph 3 of the Constitution which allows to adopt new law without a vote. This remedy, considered as anti-democratic, marked the failure of one of the most important movements of this type in France in the 21st century. These events can be considered as a defeat of democratic system - polls show that more than 80% do not agree with changes introduced by government.

**Study 5**

**Change.** We are carrying out a study on the opinions of citizens from different European countries about the political situation in their country, taking into account the opinions of experts from different fields, political scientists and economists. In the case of Spain, recent studies underline the importance of changes in the government to promote economic and social recovery after the crisis. The experts assure that a change of government would help to increase the perception of national sovereignty and strengthen the freedoms of citizens to decide on their future. These theories are also supported by various studies in fields such as Human Resources, for example, which show that the best option for a company and the most feasible way to increase its capital is to make changes in management teams to introduce new forms of leadership and management that favour productivity and satisfaction within the company (Schaufeli, 2004).

**Stability.** We are carrying out a study on the opinions of citizens from different European countries about the political situation in their country, taking into account the opinions of experts from different fields, political scientists and economists. In the case of Spain, recent studies underline the importance of maintaining a stable government in order to favour economic and social recovery after the crisis. The experts assure that the stability of the Government would help to increase the perception of national sovereignty and strengthen the freedoms of citizens to decide on their future. These theories are also supported by various studies in fields such as Human Resources, for example, where it is demonstrated that the best option for a company and the most feasible way to increase its capital is to maintain confidence in the management teams in order to redirect crisis situations based on the experience acquired during that period, favouring productivity and satisfaction within the company (Schaufeli, 2004).

**Appendix 2**

**Exploratory variables used in the study and results**

**Study 1**

**Predisposition to change.** An adaptation of the Organizational Change Skills Scale (Rabelo, Ros &Torres, 2004) was done in order to measure attitudes towards the social and political changes in general: E.g.: *It would take me a long time to adapt to the new elements introduced by the change/ The pressure for change from the government is causing me discomfort.* Participants rated their predisposition to change on twelve items (*α*= .62) on a 7-point-scale (from 1= Not at all, to 7 =Absolutely). One item was excluded to improve the reliability of the scale. The ANOVA yield no effects of the manipulation on this variable, *F*(2,146) = .124 *p*= .884 *η^2^* = .002.

**Need For closure.** (Webster & Kruglanski, 1994) This scale was found to predict important social of cognitive processes (Webster & Kruglanski, 1998): *“In case of doubt, I prefer to make an immediate decision, regardless of which one it is”/”I've never been late for work or a date”.*Participants rated this scale on sixteen items on a 6-point-scale(from 1= Strongly disagree, to 6= Strongly agree) (α= .65).

Table S1. Mean scores and standard deviations (in parentheses) of support for each party by experimental condition.

| **DV’s** | Perception of Control | Lack Of Control | Neutral | Total |
| --- | --- | --- | --- | --- |
| Main opposition party (traditional) | 23.16a (29.79) | 10.20^a^ (19.59) | 15.68 (24.62) | 15.87 (25) |
| Party in the Government | 27 (27.73) | 19.37 (25.24) | 27.10 (26.88) | 24.33 (26.63) |
| New Left Wing Party | 35.45(36.44) | 33.05(34) | 34.37 (34.24) | 34.27 (34.56) |
| New Liberal Party | 23.82(29.32) | 23.94 (27.41) | 18.28 (24) | 21.86 (26.76) |

*Note:* Significant results are marked as: ^a^ *p*<.05

The lack of government during that period was blocking political decisions, therefore we decided to manipulate the general political efficacy to see if this factor would moderate the effects of control on voting intentions. We predicted that the negative effects of low control on support for the traditional party will occur mainly when low political efficacy is emphasized.

**Study 2**

**System Justification.** It was measured as in Study 1 (Jost and Banaji, 1994), however in this case the reliability of the scale was too low (α= .26), therefore we excluded it from the analyses.

**Identification with other countries in recession.** With this scale we tried to measure the proximity towards countries that were also in a situation of economic crisis as Spain, such as Greece, Italy or Ireland. It consists of 3 items (from 1=not at all to 7= totally) that refer to different forms of proximity to the population of these countries, i.e. *I feel close to them /I feel identified with them / I feel united to them* (α =.84).

No significant effects were found on any other variables (System Justification & Europe Identification), all *Fs*<1, ns.

**Results**

There are significant results regarding the interaction of the political efficacy with the personal control measure; Control*Political efficacy *F* (1,88) *=* 5.09 *p*= .02 η^2^ = .057

Table S2. Mean scores and standard deviations (in parentheses) for support for the different political parties by experimental condition.

|  | High Control | | Low Control | |
| --- | --- | --- | --- | --- |
|  | High Political Efficacy | Low Political Efficacy | High Political Efficacy | Low Political Efficacy |
| Party in the Government | 20 (25.55) | 35.07 (28.95) | 15 (22.7) | 15.87 (27.1) |
| Main opposition party | 44.54 (78.17) | 32.72 (27.30) | 24.01 (26.65) | 20.63 (18.87) |
| New Party: Right Wing | 28.78 (25.35) | 40.7 (29.32) | 26.51 (30.05) | 27.46 (28.14) |
| New Party: Left Wing | 37.57 (36.02) | 26.28 (30.92) | 33.18 (36.62) | 35.87 (30.73) |

**Study 3**

Table S3.Mean scores and standard deviations (in parentheses) for support for the different political parties by experimental condition.

|  | High Control | | Low Control | | |
| --- | --- | --- | --- | --- | --- |
|  | High Agency | Low Agency | High Agency | Low Agency |  |
| Traditional Party (Government) | 25.80 (30.74) | 17.77 (23.72) | 17.84 (26.56) | 31.40 (34.08) | |
| Traditional Party Opposition | 28.90 (21.53) | 34.94 (25.88) | 27.74 (20.11) | 28.50 (27.82) | |
| New Right Wing Party | 33.47 (23.73) | 27.47 (26.97) | 27.64 (29.07) | 33.77 (33.42) | |
| New Left Wing Party | 30.52 (33.65) | 39.09 (33.52) | 36.76 (32.99) | 26.22 (29.91) | |

**Study 4**

**System justification.** We used eight-item scale developed by Kay and Jost (2003), α = 0.80. Participants were giving their answers on a scale from 1 (I strongly disagree) to 7 (I strongly agree).

**Opinion on the new reform.** We asked participants whether they agree that changes in labor law introduced by government were unfavorable for French society and whether, in their opinion, failure of protests affected ability of French people to successfully attain their goals (two yes/no questions).

**Hope for change***.* To measure hope for political change we used an item “I hope there will be a political change in France”. Participants answered on a scale from 1 (I strongly disagree) to 7 (I strongly agree).

**Hopes for positive future of the country.** To measure participants’ hopes for country future we used an item “My hopes for future of France are positive”. Participants answered on a scale from 1 (I strongly disagree) to 7 (I strongly agree).

**National identity***.* To measure national identity we used 2 items (“I am happy to be French” and “Being French is an important part of how I perceive myself”, α = 0.81) adapted from Leach et al. (2008). Participants answered on a scale from 1 (I strongly disagree) to 7 (I strongly agree).

**Need for change***.* We asked participants to indicate whether, in their opinion, French system requires a profound change using single yes/no question.

**Results.** We checked whether control manipulation influenced exploratory measures used in the study. We found no effects of the manipulation on perceived collective efficacy (*F*(1, 63) = 0.39, *p = .*535, system justification (*F*(1, 63) = 1.18, *p = .*281), national identity measure (*F*(1, 63) = 0.87, *p = .*353), nor need for change (*B* = -.29, *SE* = 0.63, *p = .*647, *OR* = 0.75). We didn’t find effects of control manipulation on item measuring hope for political change (*F*(1, 63) = 0.29, *p = .*590) and for hope for positive future of the France either (*F*(1, 63) = 0.19, *p = .*664). We also measured opinions on the new reforms using two questions. We found out that 68% of participants agreed that changes in labor law introduced by government were unfavorable for French society and 49% of participants believed protests affected ability of French people to successfully attain their goals. We checked whether control manipulation influenced participants’ opinions on these topics. We didn’t find influence of control manipulation on these measures either (*B* = -0.15, *SE* = 0.54, *p = .*781, *OR* = 0861; *B* = .75, *SE* = 0.50, *p = .*137, *OR* = 2.11).

**Group efficacy.** We measured group efficacy with the same items as in Study 3 (α = 0.87).

**Perceived control scale**. To check whether participants’ sense of control was restored at the end of the study we included 3-item scale of personal control (α = 0.84) adapted from Greenaway et al. (2013). The items were “I am in control of my life”, “I am free to live my life how I wish”, “My life is determined by my own actions”. Participants answered on a scale from 1 (I strongly disagree) to 7 (I strongly agree).

**Results**

***General voting intentions***

We found no main effect of control manipulation on general voting intentions (*M*_1_ = 80.00, *M*_2_ = 87.74, *F*(1, 63) = 1.03, *p = .*315, *η*² = .02). However, as some studies suggested that under threat to control people rely more strongly on information about collective agency (e.g. Stollberg et al., 2015) we explored whether there is an interaction effect between control manipulation and perceived ingroup efficacy (French people) in this study. We indeed found a significant interaction in this direction (*b* = -13.49, *SE* = 6.10, *p = .*030, *R*^2^ = 0.06). Specifically, participants’ decision about voting depended on perceived collective efficacy only for participants in the low control condition (*b* = 10.02, *SE* = 3.79, *p = .*010), but not for participants in the high control condition (*b* = -3.46, *SE* = 4.78, *p = .*471). Looking at the results differently, the difference between the two control conditions was significant only for those participants who scored low (-1 SD, *b* = 24.21, *SE =* 11.30, *p = .*036), but not high (+1 SD, *b* = -11.83, *SE =* 11.39, *p = .*302) on collective efficacy scale. Respectively, among those participants who believed ingroup efficacy is low, the ones whose sense of control was threatened were less likely to vote than participants in the high control condition. The results are presented on Figure 4.


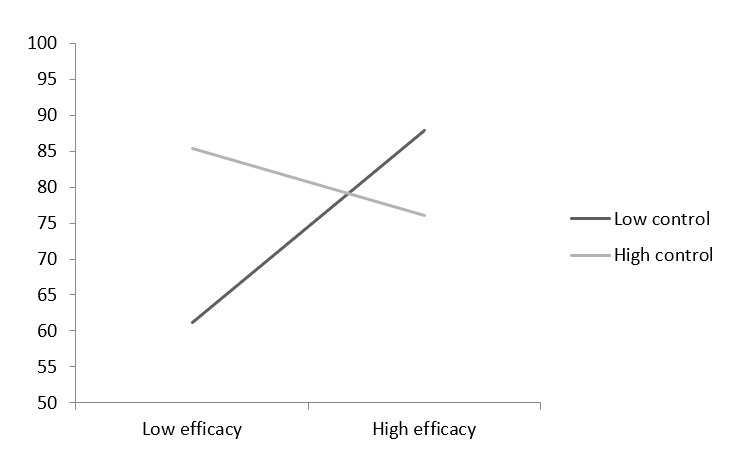


**Fig 1** *Interaction of control manipulation and perceived efficacy of French people on voting intentions in legislative elections.*

***Control restoration scale***

According to group-based control model, membership in social groups may restore people's sense of global control (e.g. Fritsche et al., 2013). Therefore, we checked whether giving participants possibility to identify with their ingroup and to engage in collective activity may restore participants' sense of perceived control over their lives. Indeed, at the end of the study no significant differences between two groups could be found (*M*_1_ = 4.58, *M*_2_ = 5.01, *F*(1, 63) = 1.49, *p = .*227, *η*^2^ = 0.02), although just after control manipulation participants in low control condition declared they felt less in control over their lives compared to high control participants.

Table 4. Mean scores and standard deviations (in parentheses) for the main dependent variables by experimental condition.

| **DV’s** | High control | Low Control | Total |
| --- | --- | --- | --- |
| Support to the Parties: |  |  |  |
| Main opposition party | 27.66(28.32) | 17.62 (21.74) | 22.32 (25.38) |
| Party in the Government | 30.18 (28.61) | 29.68 (25.43) | 29.92 (26.79) |
| New party: Left Wing | 33.69 (30.91) | 37.38 (32.81) | 35.65 (31.78) |
| New party: Center | 42.25 (32.32) | 35.63 (30.32) | 38.73 (31.25) |
| Old party: Right wing | 7.12 (15.42) | 9.92 (21.77) | 8.61 (19.00) |

**Study 5**

**Results**

Table 5 Support for the different political parties by experimental conditions

|  | High Control | | Low Control | | |
| --- | --- | --- | --- | --- | --- |
|  | Change | Stability | Change | Stability |  |
| Government Party | 30.39 (31.92) | 29.93 (36.09) | 19.43 (26.98) | 25.12 (27.18) | |
| Main Opposition Party | 29.41 (23.06) | 23.20 (23.27) | 30.25 (23.09) | 31.66 (28.48) | |
| Right-Wing Party (new) | 35.68 (29.50) | 30.93 (29.24) | 28.05 (28.27) | 36.23 (24.67) | |
| Left-Wing Party (new) | 25.81 (33.10) | 27.80 (34.23) | 36.91 (34.01) | 25.86 (29.97) | |
